# A Multi-Task Deep Learning Framework for Characterizing Beating Behavior and Synchrony in Cardiomyocyte Clusters

**DOI:** 10.3390/bioengineering13070742

**Published:** 2026-06-25

**Authors:** Tianxin Wang, Xinjie Liu, Fangshuo Zhang, Qianwen Guo, Xiaoyu Li, Yuanyuan Sun, Jingjing Xu

**Affiliations:** 1 School of Integrated Circuits, Shandong University, Jinan 250100, China; 202300180078@mail.sdu.edu.cn (T.W.); 202412491@mail.sdu.edu.cn (F.Z.); 2 School of Software, Shandong University, Jinan 250100, China; 202300180116@mail.sdu.edu.cn; 3 State Key Laboratory for Innovation and Transformation of Luobing Theory, Key Laboratory of Cardiovascular Remodeling and Function Research of MOE, NHC, CAMS and Shandong Province, Department of Cardiology, Qilu Hospital of Shandong University, Jinan 250012, China; guoqianwen10569@qiluhospital.cn; 4 School of Clinical Medical Sciences, Clinical Medicine, Cheeloo College of Medicine, Shandong University, Jinan 250012, China; 202400411005@mail.sdu.edu.cn

**Keywords:** cardiomyocyte cluster, deep learning, cluster-level segmentation, pixel difference method, beating performance characterization, beating synchrony

## Abstract

Beat-level synchrony among cardiomyocyte clusters is a critical indicator of cardiac electromechanical function. Traditional invasive approaches have substantial limitations, and conventional computer vision methods are poorly suited for resolving densely packed, adherent clusters. To address these challenges, we developed an analysis framework to characterize the beating characteristics of cardiomyocyte clusters from microscopic imaging data. Specifically, we propose CardioSegNet, a multi-task deep learning model that combines attention mechanisms with three prediction heads (semantic segmentation, contour detection, and distance transform), followed by a watershed algorithm to achieve high-accuracy cluster-level segmentation of cardiomyocyte clusters. The Pixel-Difference method is applied to extract time-series beating signals from each segmented cluster and compute several dynamic parameters, including beating amplitude, period, frequency, and the Beat Rate Irregularity (BRI). We further introduce PeriodAwareNAPTD*_ij_* to quantify the beating synchrony among different clusters. Our experimental results show that CardioSegNet achieves a Dice coefficient of 0.8868 and an HD95 of 93.02 µm on an independent test set, demonstrating strong segmentation performance. The cardiomyocyte populations are not uniformly globally synchronized; rather, they consist of multiple local subgroups with high internal synchrony, and the degree of synchronization between clusters is positively correlated with their physical distance. This label-free analytical pipeline provides an efficient tool for myocardial function evaluation and cardiotoxicity screening in vitro.

## 1. Introduction

Cardiomyocytes serve as the fundamental contractile units responsible for the heart’s continuous pumping activity. In most circumstances, these cells do not exist as isolated entities; instead, they interconnect to form dense clusters. The rhythmic beating of these clusters is a key indicator of cardiac electromechanical coupling efficiency and overall tissue functional integrity [1,2,3]. Therefore, quantifying the rhythmic properties, beating dynamics, and network synchrony of cardiomyocyte clusters provides critical insights into the mechanical contractile function of myocardial tissue [3]. It is essential for basic cardiovascular research, the development of in vitro disease models, and translational medicine. It is also highly valuable for efficient drug cardiotoxicity screening and early detection of cardiac dysfunction.

Conventional assessment of cardiac tissue function has relied primarily on electrophysiological, optical, and mechanical approaches, including microelectrode arrays (MEAs), patch-clamp recordings, and fluorescent probe-based optical mapping [4,5,6]. However, many of these techniques depend on exogenous dyes and impose considerable experimental overhead and are susceptible to photobleaching and phototoxicity. These limitations restrict their applicability to sustained, label-free analysis of living cells [7]. Therefore, non-invasive characterization of cardiomyocyte beating is more practical and broadly applicable, most commonly achieved by analyzing cardiomyocyte image datasets. For example, the Pulse platform proposed by Maddah et al. [8] divides each frame of cardiomyocyte images into fixed-size, non-overlapping blocks and extracts the average beating signal within each block by calculating the correlation coefficient between adjacent frames. Although this method is automated, it cannot identify spatially distinct cell units. A single block may contain multiple asynchronous cell clusters, leading to signal averaging and loss of local heterogeneity. In recent years, convolutional neural networks have greatly improved segmentation capabilities in medical and biological imaging, providing effective tools for cell boundary delineation and object localization. Thus, several studies have attempted to use deep learning to characterize cardiomyocyte beating. Ahmadzadeh et al. employed a fully convolutional network (FCN) to segment cardiomyocyte nuclei from digital holographic microscopy images, enabling single-cell beating signal extraction [9]. However, this method relies heavily on specialized imaging equipment and is limited to single-cell characterization, making it difficult to generalize to routine in vitro culture settings. As a result, these methods struggle to extract stable and reliable contraction features from multicellular networks, which directly limits accurate assessment of cardiomyocyte cluster-level beating dynamics [10]. Therefore, a novel computational framework is needed to address the challenges of segmenting, tracking, and temporally characterizing complex cardiomyocyte clusters.

In this paper, we present a robust, label-free, video-based algorithm to observe unlabeled cardiomyocyte cluster networks and quantitatively analyze their beating synchrony at multiple scales. This method enables high-precision segmentation, extraction of temporal signals, and comprehensive assessment of network synchrony in densely clustered cardiomyocyte networks, providing an effective tool for in vitro cardiac function assessment, drug screening, and cardiac disease modeling. The major contributions of this study are as follows:

1. We propose CardioSegNet, a multi-task deep learning model that integrates an attention mechanism and three prediction heads (semantic, contour, and distance transform). The multi-branch outputs are then processed via a watershed algorithm to separate adhered clusters into distinct instances, each representing a functional cell cluster.
2. We apply the reference-frame pixel differencing method to quantify pixel-level changes in every segmented cluster instance over time and then extract the beating characters.
3. Using the extracted beating curves, we developed a quantitative approach to translate local contraction dynamics into a measure of cell cluster synchrony.

## 2. Materials and Methods

### 2.1. Dataset

#### 2.1.1. Materials

Gelatin coating solution (Cat No. PB180535, Procell, Wuhan, China), Collagenase, Type II (Cat No. LS004177, Worthington, OH, USA), Newborn Calf Serum (Cat No. 22012-8612, Tianhang Biotechnology Co., Ltd., Tongxiang, China), Fetal Bovine Serum (Gibco, Waltham, MA, USA), Dulbecco’s Modified Eagle Medium (Gibco, USA), 5-BRDU (Cat No. 19-160, Sigma-Aldrich, Burlington, MA, USA), Penicillin-streptomycin (Gibco, USA), D-Hank’s Balanced Salt Solution (Procell System).

#### 2.1.2. Isolation and Culture of Rat Neonatal Cardiomyocytes

Neonatal Sprague–Dawley rats (1–3 days old) were sterilized by immersion in 75% ethanol. Under aseptic conditions, the chest was opened along the left sternal border to expose the heart. The heart was gently grasped with ophthalmic forceps, and the ventricular tissue was rapidly excised using curved scissors, then immediately placed in ice-cold D-Hank’s solution. The ventricles were transferred to a clean Petri dish and minced into approximately 1 mm^3^ fragments using ophthalmic scissors. The minced tissue was transferred to a conical flask and digested with collagenase II at 37 °C under gentle agitation (100 rpm) for 1 h. After digestion, the supernatant containing dissociated cells was carefully collected into a fresh centrifuge tube, and an equal volume of culture medium was added to terminate the enzymatic reaction. The cell suspension was centrifuged, the supernatant was discarded, and the pellet was resuspended in 10 mL of complete culture medium. The cell suspension was filtered through a 100 μm cell strainer and transferred to a culture flask. Fibroblasts were removed by differential adhesion. The purified cardiomyocytes were seeded onto gelatin-coated six-well plates at a density of 1 × 10^6^ cells per well. To inhibit fibroblast proliferation, 5-BRDU was added to the cardiomyocyte culture at a final concentration of 0.1 mM. After 48 h of culture, cells were imaged. The animal study protocol was approved by the Ethics Committee of Qilu Hospital of Shandong University (approval number: DWLL-202610005, approval date: 1 January 2026) and was in line with the Guidelines for the Care and Use of Laboratory Animals published by the National Institutes of Health.

#### 2.1.3. Data Acquisition

We used a biological inverted microscope (Nikon, NI-U, Yokohama, Japan) to observe rat cardiomyocytes and to acquire images and record videos of beating cardiomyocytes, totaling 574 images and 100 videos. To simulate routine laboratory observation settings and strengthen method robustness, we prepared samples at varying cell densities and acquired data at 200× and 400× magnifications. Of these, 97 images and 81 videos were captured at 200×, and 477 images and 12 videos at 400×.

#### 2.1.4. Data Annotation

We annotated all images using Labelme 5.9.1 (Kentaro Wada, GPL-3.0) in a Conda virtual environment. Labelme is an open-source annotation tool built on Python 3.9 and Qt 5.14, actively maintained on GitHub. To ensure consistent annotation quality, we adopted the following rules: (1) small, regularly shaped round cells were identified as monocytes and excluded from annotation; (2) excessively small cells were regarded as dead and excluded; (3) for cells with pseudopod extensions or blurred membrane edges, we traced the visually discernible boundary as the annotation contour; (4) when multiple cells adhered closely and individual boundaries were indistinguishable by eye, the entire cluster was annotated as a single unit.

Each visually separable cell cluster was delineated as an independent polygon, resulting in cluster-level annotations. From these annotations, we derived three types of training labels for the multi-task learning framework: (1) Semantic segmentation labels: binary masks obtained by merging all polygons; (2) Contour detection labels: cluster boundaries extracted via morphological gradient operations (dilation minus erosion) on each mask; (3) Distance transform labels: computed using cv2.distanceTransform on each mask, then merged by taking the maximum value at each pixel location.

**Figure 1:**
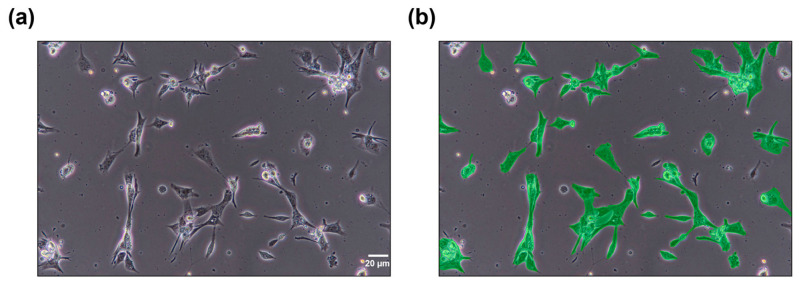
A cardiomyocyte image and its ground truth. (**a**) Original image; (**b**) Annotated mask shown in green.

We split the annotated dataset into training, test, and validation sets at an 8:1:1 ratio, yielding 458 training images, 59 test images, and 57 validation images. All ground truth masks were generated through manual annotation. Figure 1 illustrates representative original cardiomyocyte images and their segmentation examples from the dataset.

### 2.2. CardioSegNet

#### 2.2.1. Network Architecture

**Figure 2:**
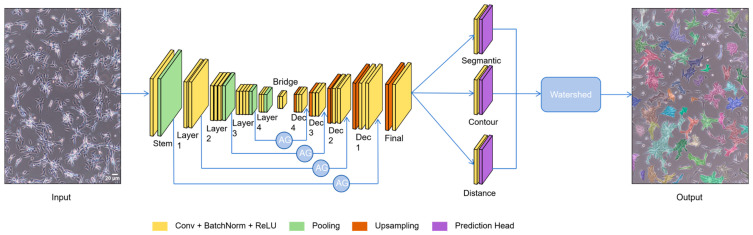
CardioSegNet architecture.

To achieve accurate cardiomyocyte segmentation, we propose a multi-task deep learning network called CardioSegNet. Built upon the U-Net [11] encoder–decoder architecture, CardioSegNet integrates residual learning and attention mechanisms, and jointly optimizes three parallel task branches for semantic segmentation, contour detection, and distance transform prediction. The overall architecture is illustrated in Figure 2.

#### 2.2.2. Encoder Module

To extract discriminative multi-scale features, we adopt a ResNet-50 [12] pretrained on ImageNet as the backbone. This encoder captures both low-level visual cues (e.g., texture and shape) and high-level semantic context of cardiomyocytes. Specifically, the input image $I\in R^{H\times W\times 3}$ first undergoes initial downsampling via a 7 × 7 convolution and max-pooling layer. The feature maps then pass through four residual stages (Stages 1–4), producing feature maps $F_{1},F_{2},F_{3},F_{4}$ at downsampling rates of 4×, 8×, 16×, and 32×, respectively. These hierarchical features retain fine spatial details at lower levels and contain rich semantic abstractions at higher levels, providing a solid basis for precise segmentation.

#### 2.2.3. Decoder Module

The decoder follows a U-Net-style architecture that progressively upsamples feature maps to restore spatial resolution. Skip connections bridge the encoder and decoder to recover fine-grained spatial details. Starting from $e_{4}$, the decoder comprises four blocks that sequentially produce intermediate features $\left\{d_{4},d_{3},d_{2},d_{1}\right\}$. Each block first applies bilinear upsampling (×2) to the input, concatenates it with the corresponding encoder feature map via the skip connection, and then refines the fused features through two successive 3 × 3 convolutions, each followed by batch normalization and ReLU.

#### 2.2.4. Attention Gate

Unlike the standard U-Net that directly concatenates encoder and decoder features, we incorporate Attention Gates (AGs) [13] into the skip connections to suppress task-irrelevant background activations and enhance the decoder’s focus on target regions. In each AG, the decoder feature serves as the gating signal and the encoder feature as the skip signal. Both are projected via 1 × 1 convolutions, summed, and passed through ReLU activation. A subsequent 1 × 1 convolution followed by a Sigmoid function produces a spatial attention weight map α, which is applied to the encoder feature through element-wise multiplication. This mechanism effectively filters out background noise and directs the network’s attention toward cardiomyocyte regions. The attention-weighted encoder feature is then concatenated with the upsampled decoder feature along the channel dimension and refined through convolutional layers, progressively restoring the spatial resolution to match the original input.

#### 2.2.5. Multitask Prediction Heads

Cardiomyocytes exhibit complex morphologies with often indistinct boundaries. To enable precise boundary delineation and segmentation, CardioSegNet employs three parallel prediction heads that jointly supervise the learning process through complementary tasks:

1. Semantic segmentation head. This branch produces a probability map $P_{sem}\in [0,1{]}^{H\times W}$ to classify each pixel as foreground (cardiomyocyte) or background. While effective for general region detection, it alone struggles to separate closely adhered instances.
2. Contour detection head. This branch outputs a probability map $P_{con}\in [0,1{]}^{H\times W}$ dedicated to predicting cluster edge boundaries. Through explicit contour learning, the network better captures inter-cluster boundaries, mitigating undersegmentation caused by cluster adhesion.
3. Distance transform regression head. This branch outputs a normalized distance transform map $P_{dist}\in [0,1{]}^{H\times W}$, where each foreground pixel value represents its normalized distance to the nearest background pixel. Peak values correspond to cluster geometric centroids. This task encourages the network to encode the internal geometry of clusters, and the high-response regions in the prediction typically correspond to cluster centers, providing reliable seed points for downstream watershed-based instance separation.

#### 2.2.6. Post-Processing of the Watershed Algorithm

To characterize the beating behavior of cell clusters, we first apply connected component analysis to the semantic segmentation masks to identify spatially separated clusters. Given the practical constraints of manual annotation cost, our annotation strategy was to delineate only boundaries that were visually discernible. However, the substantial adhesion and overlap among cardiomyocyte clusters often result in a single annotated instance containing multiple cell clusters with distinct beating characteristics. In such cases, connected component analysis alone cannot reliably distinguish these clusters that are potentially meaningful for fine-grained analysis. To support more detailed exploration at the cluster scale, building on the multi-branch network outputs, we design a multi-branch feature fusion-based watershed post-processing pipeline [14] that exploits the complementary predictions from all three heads for further segmentation after the connected component analysis. The semantic prediction map is first binarized at a high threshold ($T_{sem}=0.85$) to yield a high-confidence foreground mask. The contour prediction map is binarized at $T_{cont}=0.5$ and dilated to form a boundary exclusion region. Gaussian smoothing (σ=12.0) is then applied to the distance transform map, and local maxima are detected on the smoothed surface to identify candidate seed points. To suppress false seeds, each candidate must satisfy two criteria: it must lie within the semantic foreground and must not overlap with the detected contour. A minimum Euclidean distance of 300 pixels between adjacent seeds is further enforced to guarantee a one-to-one correspondence between seeds and clusters. Using the filtered seeds as markers, the negated distance transform map as the topographic surface, and the semantic foreground mask as the watershed boundary constraint, we execute the watershed algorithm and then complete the cluster labeling. The resulting instance labels are then renumbered consecutively. The watershed algorithm substantially mitigates the limitations of manual annotation by segmenting smaller regions, thereby enabling a more detailed study of cardiomyocyte beating. Researchers can adjust the seed point parameters of the watershed algorithm to control the degree of subdivision according to their specific analytical needs, and the resulting subregions serve as the basic units for subsequent beating signal extraction.

### 2.3. Split Effect and Stability

All experiments were conducted on a Linux workstation with an Intel Xeon Gold 6248R CPU and a single NVIDIA A40 GPU, using PyTorch 2.10.0 and Python 3.10.19. We comprehensively evaluated the segmentation performance of CardioSegNet, U-Net, and DeepLabV3+ on our custom dataset (Section 2.1). All three models followed a unified training protocol. To mitigate the effect of imbalanced cell density distributions, we employed stratified 5-fold cross-validation based on foreground density binning. During training, 8 random 512×512 crops were sampled from each training image per epoch, with a batch size of 16. The maximum number of training epochs was set to 100. We used AdamW as the optimizer with an initial learning rate of $1\times 10^{-4}$ and a weight decay of $1\times 10^{-4}.$ Early stopping with a patience of 30 epochs was applied to prevent overfitting.

To evaluate the segmentation performance and stability differences between our method, the U-Net model, and the DeepLabV3+ model. We adopt the Dice similarity coefficient (DSC), which quantifies the overlap between predicted and ground truth regions [15]. It is defined as:

(1) $$\begin{array}{c} DSC=\frac{2\;TP}{2\;TP+FP+FN} \end{array}$$

where *TP*, *FP*, and *FN* are the numbers of true positive, false positive, and false negative detections, respectively.

CardioSegNet achieved the best validation Dice scores of 0.9140, 0.9015, 0.9212, 0.9140, and 0.9189 across the five folds, yielding a mean of 0.9139±0.0076. In comparison, U-Net and DeepLabV3+ obtained mean validation Dice scores of 0.8936±0.0079 and 0.8750±0.0102, respectively. The notably lower inter-fold standard deviation of CardioSegNet indicates that its performance gains are consistently maintained across different training–validation partitions, whereas the other two models exhibit greater sensitivity to data splits. The experimental results demonstrate that the CardioSegNet model has significant advantages in segmentation stability and generalization. Table 1 shows the Dice parameter results of each model inferred on the test set. CardioSegNet outperforms U-Net by 1.16% and DeepLabV3+ by 2.83% in Dice, confirming its superior capability in accurately segmenting cardiomyocyte regions.

Cardiomyocytes exhibit irregular and heterogeneous morphologies, and microscopic images of these cells often present challenges such as uneven contrast, blurred edges, and inter-cell adhesion. Conventional models tend to produce severe segmentation deviations in these common challenging regions. Since this task demands high boundary precision in cell segmentation, we introduce the 95th-percentile Hausdorff Distance (HD95) [16] to specifically evaluate the boundary accuracy of each model, which can reduce the impact of outliers on the Hausdorff distance. Let ∂P and ∂G denote the predicted and ground truth boundary point sets, respectively. The directed surface distance from ∂P to ∂G is defined as:

(2) $$\begin{array}{c} d(\partial P,\;\partial G)=\{\underset{g\in \partial G}{\min}\|p-g{\|}_{2}:p\in \partial P\} \end{array}$$

HD95 is defined as the maximum of the 95th percentile distances in both directions, measuring the maximum deviation between the predicted boundary and the ground truth boundary. A lower HD95 value indicates higher alignment accuracy between the predicted contour and the ground truth contour:

(3) $$\begin{array}{c} HD95(P,G)=\max({perc}_{95}d(\partial P,\partial G),{perc}_{95}d(\partial G,\partial P)) \end{array}$$

HD95 was computed on the binary semantic foreground mask for each image. As shown in Table 1, CardioSegNet achieves an HD95 of 93.02 µm, a reduction of 6.14 µm compared to U-Net (99.16 µm) and 20.13 µm compared to DeepLabV3+ (113.15 µm), corresponding to decreases of 6.19% and 17.79%, respectively. This indicates that the model achieves lower average errors in high-quantile boundary error control, demonstrating higher precision.

**Figure 3:**
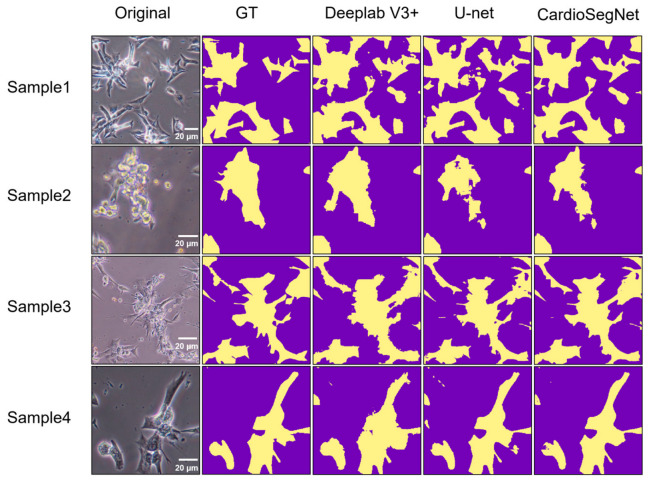
Visual Comparison of Segmentation Performance Among the Three Models.Yellow indicates cardiomyocyte regions (foreground); purple indicates background.

As shown in Figure 3, the segmentation mask produced by CardioSegNet most closely resembles the original image and the ground truth (manual annotation), featuring the smoothest edges and the fewest misclassified pixels. These results indicate that our model effectively suppresses boundary irregularities and distortions when handling the complex spatial structures of cardiomyocyte clusters, reducing false boundary predictions at local edges and demonstrating superior boundary segmentation accuracy.

To further compare CardioSegNet with a recent state-of-the-art foundation model for cell segmentation, we evaluated Cellpose-SAM using the official cpsam_v2 pretrained model on our cardiomyocyte cluster test dataset in a zero-shot setting. To adapt Cellpose-SAM to the morphology and scale of our cardiomyocyte cluster dataset, we tuned its inference-related parameters on the test set (flow_threshold = 0.8, cellprob_threshold = 0.5, min_size = 300, diameter = 100). As shown in Table 1, Cellpose-SAM achieved a Dice coefficient of 0.8026 ± 0.1139 and an HD95 of 135.82 µm. CardioSegNet showed an 8.4% higher Dice coefficient and a reduction to zero-shot of 42.8 µm, corresponding to a decrease of 31.51%. These results indicate that CardioSegNet achieved higher segmentation accuracy in this specific application scenario. In terms of model complexity, CardioSegNet contains 34.4 M parameters, which is approximately 11% of the 304.6 M parameters of Cellpose-SAM. This smaller model size makes it more suitable for deployment on standard laboratory workstations and for integration into practical cardiomyocyte video analysis workflows.

### 2.4. Analysis Methods and Evaluation Metrics of Cardiomyocyte Pulsation

#### 2.4.1. Pulse Signal Extraction—Pixel Differential Method

Using the CardioSegNet segmentation model described above, we first segment the initial frame of each video to generate a binary mask. The mask defines the region of interest (ROI), which can exclude the influence of background noise. Most importantly, it constrains the spatial scope of subsequent computations—all subsequent operations are performed only in regions where the mask value is “1”, thereby reducing computational complexity. The output mask is then processed by the watershed algorithm to delineate individual cell clusters, each of which is assigned a unique instance ID. Within the ROI of each identified cell cluster, we extract temporal difference signals [17,18]. For the c-th cell cluster, let $\Omega_{c}$ denote its ROI and $G_{t}(x,y)$ denote the grayscale image at time t. The grayscale intensity sums over the ROI at time t is defined as:

(4) $$\begin{array}{c} P_{c}(t)=\sum_{(x,y)\in \Omega_{c}}G_{t}(x,y) \end{array}$$

A resting-state frame is then selected as the reference frame $I_{ref}$, serving as the grayscale baseline. Based on this, for the frame at time t, the raw beating signal is constructed by computing the pixel-wise absolute grayscale difference with respect to the reference frame, thereby quantifying the magnitude of morphological change in the cell relative to the resting state at that time point and capturing the temporal beating dynamics of cardiomyocytes [17,19]. To standardize beating intensity measurements across cell clusters of different sizes, we normalize the absolute grayscale difference by the number of pixels within the corresponding ROI. Denoting the pixel count as $N_{c}=|\Omega_{c}|$, the normalized signal is given by:

(5) $$\begin{array}{c} S_{c}(t)=\frac{\left|P_{c}(t)-P_{c}(t_{ref})\right|}{N_{c}} \end{array}$$

**Figure 4:**
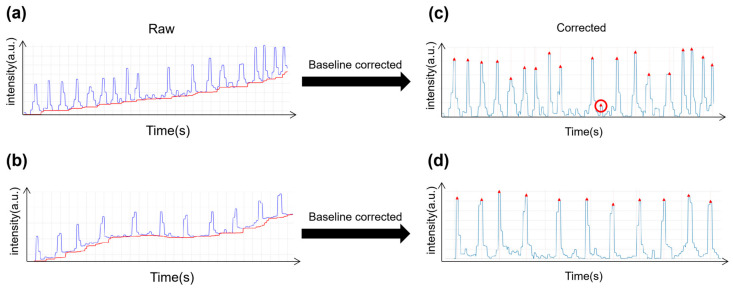
Comparison before and after baseline correction. (**a**) Beating output curve of Instance 1 before correction; (**b**) Beating output curve of Instance 2 before correction; (**c**) Instance 1 after correction, with an inappropriate threshold leading to peak misidentification; (**d**) Instance 2 after correction with a properly adjusted threshold, showing no misjudgment.

As shown in Figure 4a,b, although the beating time points can be observed from the output curves, the images generally exhibit baseline drift, indicating instability in the optical system and highlighting the limitation of using a fixed reference frame. Therefore, we adopt a dynamic baseline correction method that automatically tracks the slowly changing baseline while preserving the rapid variation features of beats, achieving the effect of eliminating long-term drift. Peak detection is then performed on the corrected signal to identify key time points in each beating cycle and to extract quantitative beating parameters. Meanwhile, identifying beating peaks requires an appropriate threshold. As indicated by the circles in Figure 4c, an improperly set threshold may misclassify spikes in the acquired video as beating peaks, leading to erroneous results. After setting a proper threshold, as shown in Figure 4d, the algorithm identifies only clear peaks as beating events. This substantially reduces misclassification and improves the accuracy of the algorithm.

#### 2.4.2. Beating Performance Metrics

We define each cardiomyocyte beat as comprising three phases: rest, contraction, and relaxation. As listed in Table 2, several parameters are defined to quantitatively characterize the beating behavior. Amplitude measures the intensity of a single beat and is defined as the signal difference between the main peak and the preceding valley. A larger amplitude corresponds to a more pronounced grayscale change, indicating a stronger contractile response. The beating period describes the rhythmicity of cell contractions and is defined as the time interval between two consecutive main peaks. Beating frequency is accordingly defined as the number of beats per minute (BPM) [20,21]. To further characterize the temporal dynamics of individual beat waveforms, we define two additional parameters: rise time and decay time. Rise time is the duration from the preceding valley to the main peak, reflecting the process of the beat establishment phase. Decay time is the duration from the main peak to the subsequent valley, reflecting the recovery process of the relaxation phase [20,21]. Furthermore, to characterize the overall duration and broadening features of a single beat, we introduce the half-amplitude width. For the i-th beating cycle, the half-amplitude level is defined as:

(6) $$\begin{array}{c} H_{i}=\underset{c}{\tilde{S}}(t_{i}^{-})+\frac{A_{i}}{2} \end{array}$$

Let the signal intersect the half-amplitude level on the rising and falling segments of the main peak at time $t_{1/2}^{↑}$ and $t_{1/2}^{↓}$, respectively. The half-amplitude width is then defined as:

(7) $$\begin{array}{c} {\mathrm{TIBD}}_{50,i}=t_{i,1/2}^{↓}-t_{i,1/2}^{↑} \end{array}$$

A central objective of this study is to examine the synchrony and variability of beating across different cell clusters. To quantify the irregularity of beating rhythms, we define the rhythm irregularity index as the coefficient of variation in all beating periods within a recording period [22]:

(8) $$\begin{array}{c} \mathrm{BRI}=\frac{\sigma_{T}}{\mu_{T}} \end{array}$$

where $\mu_{T}$ and $\sigma_{T}$ denote the mean and standard deviation of all beat-to-beat intervals within the recording, respectively. A larger BRI indicates more pronounced fluctuations between adjacent beating periods, suggesting lower stability of the cell beating rhythm. The above parameters can be used to characterize the overall beating dynamics and stability of a single cell during the recorded period.

To further validate the reliability of beating-parameter extraction within the proposed computational framework, we performed a manual-annotation control experiment. An experienced expert, blinded to the algorithmic outputs, manually reviewed 400 cardiomyocyte clusters from 16 sample videos, annotated their beating peak times, and counted the number of beats. Peak matching was performed using a one-to-one matching strategy with an appropriate matching tolerance window. Based on the matching results, precision, recall, and F1 score were calculated. The agreement of the matching results was further evaluated using mean absolute error, Pearson correlation analysis, and Bland–Altman analysis. The experimental results are presented in Section 3.1.

#### 2.4.3. Metrics for Cardiomyocyte Beating Synchrony

Cardiomyocytes transmit electrical signals to neighboring cells via gap junctions, producing coordinated spontaneous beating [1,23]. Having extracted the beating characteristics of each cluster, we next quantified inter-cluster synchrony to characterize the collective beating behavior of the cardiomyocyte population. Synchrony analysis was performed on each cluster in the field of view across multiple recorded samples.

We analyzed inter-cluster beating synchrony using a refined period-aware synchrony metric. Jongsma et al., in their study of beating synchronization in cultured cardiomyocyte pairs, defined latency as the time difference between the contraction instants of two cells, serving as a fundamental measure of inter-cellular synchrony [2]. Building on this concept, we generalize the single-beat latency to a statistical mean over multiple beating cycles, defined as:

(9) $$\begin{array}{c} {APTD}_{ij}=\frac{1}{N}\sum_{k=1}^{N}|t_{i,k}-t_{j,k}| \end{array}$$

where $t_{i,k\;}$ and $t_{j,k}$ denote the peak time of the *k*-th beat for cell i and cell j, respectively, and N is the total number of matched beat pairs. APTD is the multi-beat statistical mean of the latency originally defined by Jongsma et al., and directly captures the average temporal offset between the peak instants of two cells. However, APTD is an absolute time measure and does not account for differences in beating period: pairs with longer periods naturally allow greater timing offsets, which limits comparability across pairs with different beating rates. To address this, we normalize the absolute peak offset by the shorter of the two cells’ mean beating periods, converting it into a dimensionless quantity relative to the rhythm timescale. This yields a refined period-aware synchrony metric, defined as:

(10) $$\begin{array}{c} {PeriodAwareNAPTD}_{ij}=\frac{{APTD}_{ij}}{\min(T_{i},T_{j})} \end{array}$$

where $T_{i}$ and $T_{j}$ denote the mean beating periods of the cell pairs. The shorter of the two periods is used as the normalization baseline, since it corresponds to the faster-beating cluster, which has a narrower tolerance window for phase offset. This choice makes the metric more sensitive to timing deviations in beating clusters, yielding a stricter criterion for synchrony assessment. A threshold of PeriodAwareNAPTD*_ij_* ≤ 0.10 indicates that the mean peak-time offset between the two cells is less than 10% of the faster cell’s beating period and is used to identify cluster pairs with high beating synchrony. The identified high-synchronization sample examples are provided in the Supplementary Materials (Figure S1). In this study, this value was used as an operational threshold for identifying highly synchronized cluster pairs rather than as a universal biological boundary. To assess whether the choice of this threshold affected the main conclusions, we further performed a threshold sensitivity analysis at three cutoff values, 0.05, 0.10, and 0.15, as described in Section 3.4.

To further validate whether PeriodAwareNAPTD*_ij_* characterizes inter-cluster synchrony consistently with conventional signal correlation analysis, we calculated the Pearson correlation coefficient between the peak sequences of every pair of valid beating clusters within the same video and used it as a reference metric. The experimental results are presented in Section 3.3.

Based on the above method, this study developed a locally built web platform for characterizing cardiomyocyte cluster beating. The platform enables integrated analysis of raw microscopy videos and algorithm outputs. It adopts a lightweight front-end and back-end separated architecture. The back-end, implemented in Python, provides video streaming and data interfaces. It automatically matches each acquired video with its corresponding analysis results. The front-end offers video playback, time cursor synchronization, cell-level curve browsing, and interactive filtering. The specific workflow is as follows: first, load the metadata and cluster features of a single video. Then, display the corresponding beating curves of the cell clusters under a unified time reference. This allows rapid verification and comparison of cluster peak rhythms and overall heart rate metrics. The platform is mainly used for standardized result validation and reproducibility analysis. It ensures consistency in experimental data processing and result interpretation. The interface and functional examples of the platform are provided in Figure S2 of the Supplementary Materials. The source code, demonstration videos, or deployment instructions are available from the authors upon reasonable request to facilitate further understanding and reproduction of the analysis workflow.

## 3. Results and Discussions

### 3.1. Characterization of Beating Performance

**Figure 5:**
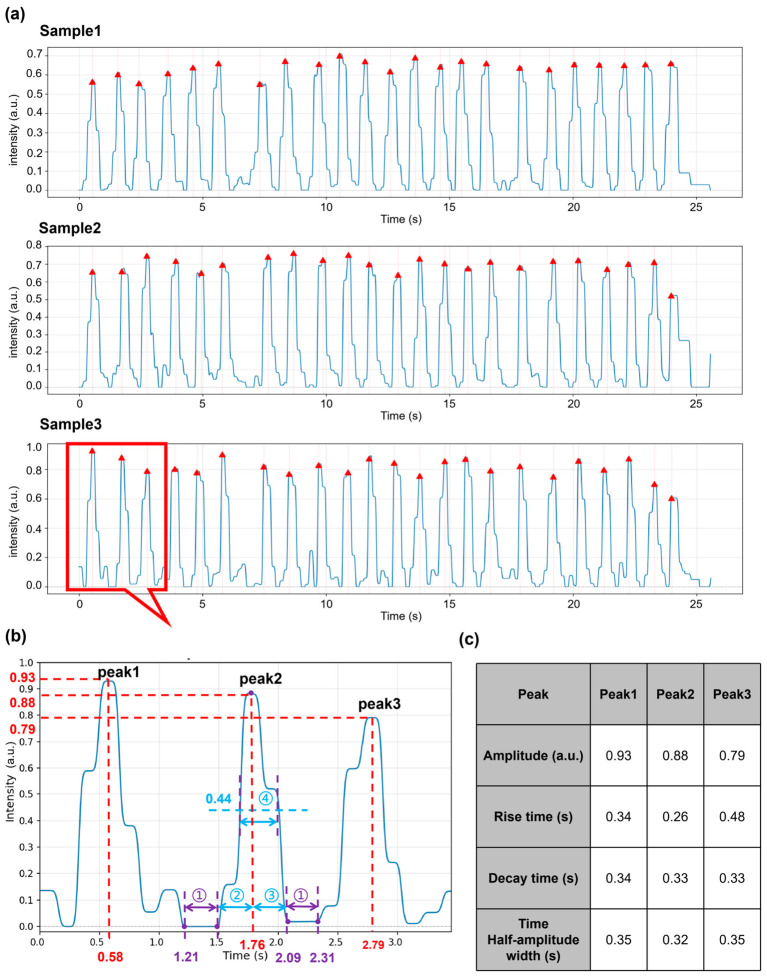
Example of cardiomyocyte beating performance characterization using CardioSegNet and the pixel difference method. (**a**) Beating curves of three cell clusters. (**b**) Beating performance analysis of a single cycle. (**c**) Performance characterization of the first three beating cycles of Sample 3.

We applied the above workflow to 16 samples (Videos S1–S16) and obtained their corresponding beating curves. Figure 5a shows the reconstructed beating curves for three representative cell clusters, and other instances are presented in the Supplementary Materials (Figure S3). Each beating cycle follows a consistent pattern, consisting of a pre-peak trough, a main peak, and a post-peak trough. The main peak represents the point of maximum contraction, where the cell deviates most from its resting state. The pre-peak trough marks the onset of contraction, and the post-peak trough marks its completion. Based on these landmarks, each cycle is divided into three phases: the resting phase, spanning from the post-peak trough of the preceding cycle to the pre-peak trough of the current cycle; the contraction phase, from the pre-peak trough to the main peak; and the relaxation phase, from the main peak to the post-peak trough. As illustrated in Figure 5b for three consecutive cycles from Sample 3: interval ① shows zero signal amplitude, corresponding to the resting phase; interval ② shows a sharp rise in contractile signal that reaches its peak over the rise time, corresponding to the contraction phase; and interval ③ shows a gradual decline back to zero, corresponding to the relaxation phase [2]. Together, these three intervals constitute one complete beating cycle. Figure 5c presents the quantified beating parameters for the first three cycles of Sample 3. Comparing these parameters across cycles provides granular insight into the beating regularity and potential abnormalities of individual cardiomyocyte clusters.

Moreover, the beating curve of each cluster encodes additional dynamic information. Using the analysis framework described in Section 2.4.2, parameters including beating period, beating rate, and beat rate irregularity were extracted for each cluster. Table 3 presents these parameters for three representative samples derived using CardioSegNet and the pixel-difference method. The beating period and beating rate showed little difference across samples, with mean values of 1.084827 s and 55.3347 bpm, respectively. The BRI of the three samples ranged from 0.180801 to 0.275082, with a mean of 0.222033. A lower BRI indicates a more regular rhythm. In general, the BRI values below 0.3 for cardiomyocyte beating are considered relatively stable. In this experiment, the BRI values of all three samples were below 0.28, and those of Sample 2 and Sample 3 were below 0.22. These results indicate that the beating rhythm of each cell cluster exhibits small fluctuations and good regularity, consistent with the physiological characteristics of normal cardiomyocyte beating. Comparison of these three parameters can be used to assess whether the rhythm of each cell cluster is normal and to evaluate the correlation of beating rhythms among clusters [10,24]. These results demonstrate that the CardioSegNet-based analysis pipeline can automatically extract multiple quantitative beating parameters, including beating period, frequency, rhythmic regularity, etc., from video data. The extracted parameters closely match the beating patterns observed in the recorded videos, confirming the validity of this approach for label-free, non-invasive analysis of cardiomyocyte beating behavior.

We performed the manual-annotation verification experiment described in Section 2.4.2. Using the manual annotations as the reference, the algorithm achieved a peak-detection precision of 82.92%, a recall of 84.10%, and an F1 score of 0.835. For beating-rate estimation, the mean absolute error between the algorithm-derived results and the manual counts was 3.732 bpm. The two measurements showed a positive correlation (Pearson r = 0.926, *p* < 0.001). Bland–Altman analysis showed that the mean bias of the algorithm relative to manual counting was 2.163 bpm, with 95% limits of agreement ranging from −9.438 to 13.764 bpm, indicating no evident systematic overestimation or underestimation. These results indicate that the proposed pixel-difference-based analysis pipeline can reliably quantify beating rate at the cluster level.

### 3.2. Overall Distribution of Synchrony

**Figure 6:**
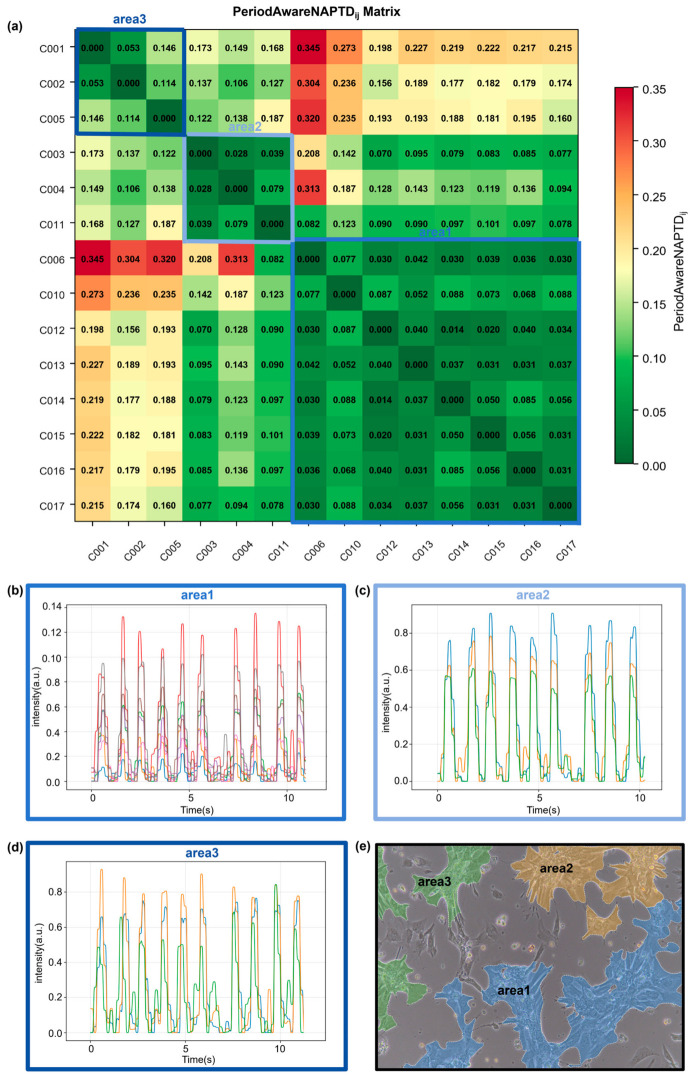
Beating synchrony analysis based on the PeriodAwareNAPTD*_ij_* matrix for a representative acquired video. (See Video S3 in the Supplementary Material for the original video.) (**a**) PeriodAwareNAPTD*_ij_* matrix; (**b**–**d**) Comparisons of beating curves within subgroups area 1, area 2, and area 3, respectively; (**e**) Schematic of spatial positions of the three subgroups.

Figure 6a shows the PeriodAwareNAPTD*_ij_* matrix for one representative sample (Video S3). The beating curves of all active cells show strong temporal alignment, reflecting high overall synchrony. Within this field of view, 14 active beating cells were identified. PeriodAwareNAPTD*_ij_* values across cell pairs range from 0.0142 to 0.3449, with a matrix mean of 0.1221. Approximately 49.45% of cell pairs satisfy PeriodAwareNAPTD*_ij_* ≤ 0.10, while only 5.49% exceed 0.25, indicating that peak-time offsets for most pairs represent only a small fraction of the shorter beating period. This confirms that the cardiomyocyte population in this sample exhibits high collective synchrony. The mean beating periods of all cells fall within 0.98–1.18 s, further indicating that the beating rates are highly uniform across cell clusters in this sample. More importantly, three functional subgroups can be identified from the matrix structure, as shown in Figure 6b–d. The first subgroup (Figure 6b), regarded as the core synchrony unit of this sample (that is, it has the strongest internal synchrony and contains the largest number of cell clusters), comprises C06, C010, C012, C013, C014, C015, C016, and C017. The internal PeriodAwareNAPTD*_ij_* values range from 0.014 to 0.088 (mean = 0.0476), well below the 0.10 threshold, confirming high synchrony among its members. The second subgroup (Figure 6c) consists of C003, C004, and C011, with internal PeriodAwareNAPTD*_ij_* of 0.0284–0.0786 (mean = 0.0488). The inter-subgroup values between it and the first subgroup fall predominantly within 0.0695–0.1433, indicating the slightly weaker coupling with the core unit. The third subgroup (Figure 6d) comprises C001, C002, and C005, with internal PeriodAwareNAPTD*_ij_* of 0.0534–0.1458 (mean = 0.1043). Its inter-subgroup values with the first subgroup rise to 0.1559–0.2265, indicating that although this subgroup maintains internal synchrony, it is largely decoupled from the core unit with a pronounced phase offset. As shown in Figure 6e, visual inspection of the recorded video confirms that cell clusters within each subgroup are tightly grouped, forming compact and distinct spatial assemblies. Meanwhile, as noted above, the internal synchrony of the first (area 1) and second (area 2) subgroups is higher than that of the third subgroup (area 3). Comparing the spatial positions of the three subgroups shows that the physical distance between the second and first subgroups is shorter than that between the third and first subgroups. This further supports that the degree of synchrony among cardiomyocyte clusters is related to their spatial physical distance to some extent.

These results indicate that the beating pattern of cell clusters does not show uniform global synchrony, but instead exhibits a clear hierarchical organization: clusters within each subgroup are more tightly synchronized, while inter-subgroup synchrony varies considerably. The elevated PeriodAwareNAPTD*_ij_* between the core unit and the third subgroup suggests that, while cardiomyocytes within the same field of view maintain a relatively high overall synchrony level, the underlying pattern more closely resembles a modular organization of locally coupled units rather than a uniform global process. This subgroup-level organization and spatial heterogeneity are consistent with previous reports [25,26]. We also observed a weak but consistent relationship between inter-cluster synchrony and physical distance within and between subgroups, which is analyzed in detail in the following section.

To further validate the effectiveness of using PeriodAwareNAPTD*_ij_* to assess the synchrony of cardiomyocyte clusters, we compared the PeriodAwareNAPTD*_ij_*-based synchrony analysis with Pearson correlation analysis based on beating peak sequences. This validation experiment was performed on 16 sample videos and included a total of 5034 cardiomyocyte cluster pairs.

The results showed a clear negative correlation between PeriodAwareNAPTD*_ij_* and the Pearson correlation coefficient of peak sequences (details are provided in Supplementary Material Table S1 and Figure S4). Across the 16 samples, the mean Pearson correlation coefficient between PeriodAwareNAPTD*_ij_* and peak-sequence Pearson correlation was −0.6295, and the mean Spearman correlation coefficient was −0.6218. A smaller PeriodAwareNAPTD*_ij_* indicates a lower average peak-time offset between two clusters, and their peak sequences are expected to show higher consistency. Conversely, a larger Pearson correlation coefficient of peak sequences indicates greater peak coincidence between two clusters and thus stronger synchrony. The observed negative correlation between PeriodAwareNAPTD*_ij_* and peak-sequence Pearson correlation supports the validity of PeriodAwareNAPTDij for assessing the synchrony of cardiomyocyte clusters. Moreover, unlike Pearson correlation alone, PeriodAwareNAPTDij is directly based on peak-time offsets and further accounts for differences in the mean beating periods of different clusters, thereby providing a more explicit time-scale interpretation while reflecting synchrony.

### 3.3. Spatial Distribution of Synchrony

**Figure 7:**
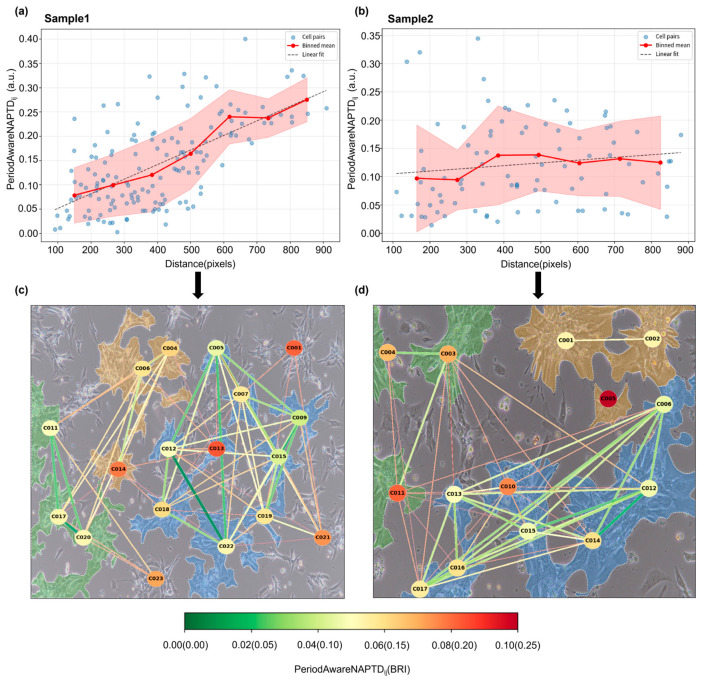
Relationship among physical distance, cluster BRI, and beating synchrony degree of cardiomyocyte clusters. (**a**,**b**) Relationship between physical distance and period-aware synchrony metric for Sample 1 and Sample 2, respectively; (**c**,**d**) Synchrony networks for Sample 1 and Sample 2. Cell clusters with the same color belong to the same subgroup. Line color indicates the synchrony degree between two clusters. Node color represents the BRI value of each cluster, which reflects the regularity of its beating rhythm.

Analysis of spatial cell locations within these subgroups showed clear grouping. To test whether cardiomyocyte signal transmission is associated with physical distance, we performed a spatial synchrony analysis. As shown in Figure 7, PeriodAwareNAPTD*_ij_* values across cell pairs at different separations showed a weak but consistent positive association with inter-cluster distance. Across all 16 samples, the Pearson correlation between inter-cluster distance and the period-aware synchrony index was r = 0.180 (11 samples reached statistical significance (*p* < 0.05)), and the Spearman rank correlation was 0.217 (11 samples reached statistical significance (*p* < 0.05), see Figure S5 and Table S2 in the Supplementary Material for related images and data). These results suggest that synchrony tended to decrease slightly as spatial distance increased. Thus, nearby clusters are more likely to beat synchronously, consistent with previous reports [26,27]. We further compared the mean spatial distances of synchronous and asynchronous cluster pairs. In Sample 1 (Figure 7a,b, Video S6), the mean distance was 297.40 pixels for synchronous pairs and 471.34 pixels for asynchronous pairs. In Sample 2 (Video S3), the corresponding values were 350.96 and 461.68 pixels. This experimental result further demonstrates that synchronized cell cluster pairs tend to be more localized to specific spatial regions rather than being randomly distributed across the field of view. Meanwhile, based on the PeriodAwareNAPTD*_ij_* matrix in Section 2.4.3, we compared the spatial positions of synchronized cell cluster pairs across multiple samples (Sample 1 and Sample 2 are shown here for illustration; see Figure S6 in the Supplementary Material for additional details). As shown in Figure 7c,d, highly synchronous samples are typically organized into several subgroups. Within each subgroup, PeriodAwareNAPTD*_ij_* values are small and clusters form compact neighboring structures; between subgroups, PeriodAwareNAPTD*_ij_* values are markedly higher, showing clear separation from intra-subgroup values. These results indicate that synchronous pairs are preferentially localized in nearby regions, rather than randomly distributed across the field of view. We further compared each cluster’s rhythm irregularity index with its synchrony strength relative to other clusters. We find that clusters with more regular intrinsic beating tend to show stronger synchrony with neighboring clusters. In contrast, clusters with unstable intrinsic rhythms behaved more independently and were less likely to establish strong coupling. These results suggest that spatial proximity supports inter-cluster synchronization, but synchrony is also shaped by intrinsic rhythm stability. Clusters with stable intrinsic rhythms are more readily synchronized and better integrated into the synchrony network, whereas rhythm-unstable clusters tend to remain weakly coupled, relatively independent oscillatory units. This pattern agrees with previous reports [28,29].

However, the small magnitudes of both correlation coefficients and the fact that five samples did not reach statistical significance indicate that physical distance alone cannot fully explain the observed synchrony patterns. It may reflect the multifactorial nature of cardiomyocyte synchronization. In addition to physical distance, inter-cluster synchrony may be influenced by the distribution and quality of gap-junctional coupling, cellular maturity, local cell density, tissue architecture, and the intrinsic rhythm stability of individual clusters. The latter was also observed in our data, as clusters with more regular intrinsic beating tended to show stronger coupling with neighboring clusters, whereas rhythm-unstable clusters behaved more independently. Moreover, the inter-cluster distances measured in this study were derived from two-dimensional microscopic images and may not fully represent the actual three-dimensional conduction paths or heterogeneous routes of signal propagation between clusters. Residual errors from image segmentation and peak detection may also introduce additional measurement noise. These factors likely weaken the distance-synchrony correlation measured from two-dimensional image data and are consistent with the weak but directionally consistent association reported here.

PeriodAwareNAPTD*_ij_* matrix-based synchrony analysis enables real-time characterization of cardiomyocyte population organization and dynamic remodeling under different external stimuli or conditioning states. In drug safety assessment, pre- and post-treatment matrix comparisons can reveal whether a compound disrupts inter-cluster synchronous beating, thereby indicating potential pro-arrhythmic risk. In hiPSC-CM culture optimization, the spatiotemporal emergence of synchronized clusters reflects both cellular maturation and the establishment of intercellular coupling. In tissue engineering, this metric can be used to evaluate how electrical stimulation or scaffold materials promote local synchrony-network formation. Importantly, the framework is not restricted to a single acquisition modality and is compatible with multiple experimental platforms, including impedance-based assays, calcium imaging, and optical motion tracking.

### 3.4. Threshold Sensitivity Analysis of the Synchrony Parameter

To further evaluate the rationality and robustness of the synchrony criterion PeriodAwareNAPTD*_ij_* ≤ 0.10, we repeated the synchrony analysis using three thresholds, 0.05, 0.10, and 0.15, on the same dataset (Videos S1–S16) and within the same set of valid beating clusters.

We quantified the proportion of cell-cluster pairs classified as highly synchronized among the 16 samples under the three thresholds (detailed results are provided in Supplementary Material Table S3). As the threshold was relaxed from 0.05 to 0.15, the proportion of cluster pairs classified as highly synchronized gradually increased. The mean proportions of highly synchronized cluster pairs within each video were 4.67%, 9.91%, and 17.26% at thresholds of 0.05, 0.10, and 0.15, respectively. This result was expected, as the absolute number of synchronized cluster pairs changes with the threshold: a stricter threshold retains only cluster pairs with highly consistent peak timing, whereas a more relaxed threshold includes more cluster pairs with slight temporal offsets but still a certain degree of synchrony. PeriodAwareNAPTD*_ij_* ≤ 0.10 indicates that the average peak-time offset between two clusters is less than 10% of the beating period of the faster cluster. For the cardiomyocyte clusters in this dataset, the mean beating period was approximately 1.0318 s, corresponding to an absolute peak-time offset of approximately 0.1 s. Relative to the time scale of a complete beating cycle, we considered a phase offset of less than 10% of the period to be a relatively strict criterion for identifying highly synchronized cluster pairs.

We also compared the identification of highly synchronized subgroups across samples under different thresholds (detailed results are provided in Supplementary Material Table S4). The subgroup structures identified under different thresholds were generally consistent. The stricter threshold of 0.05 mainly identified the core cluster modules with the strongest synchrony. When the threshold was relaxed to 0.10 and 0.15, a small number of neighboring clusters were further incorporated into existing subgroups or formed additional small synchronized subgroups. However, across all samples, the main synchronized modules did not change substantially with the threshold. The core synchrony units identified at the 0.05 threshold remained central in the results obtained at the 0.10 and 0.15 thresholds, showing a structural pattern that expanded outward from the core synchrony units. These findings indicate that the observed synchrony behavior of cardiomyocyte clusters was not artificially generated by a single threshold, but instead reflected relatively stable local modular organization.

We further compared the overall trend between synchrony and spatial distance under different thresholds (detailed results are provided in Supplementary Material Table S5) and found that the weak correlation pattern was not altered. At the threshold of 0.05, the mean distances of synchronized and asynchronous cluster pairs across the 16 samples were 282.87 pixels and 485.98 pixels, respectively. At the threshold of 0.10, the corresponding values were 315.76 pixels and 491.67 pixels, and at the threshold of 0.15, they were 325.25 pixels and 501.64 pixels. Under all three thresholds, the mean distance of synchronized cluster pairs was smaller than that of asynchronous cluster pairs, indicating that spatially closer clusters tended to exhibit higher synchrony. Therefore, the choice of the synchrony-parameter threshold did not affect this conclusion.

Taken together, these results show that although the number of highly synchronized cluster pairs changes with the PeriodAwareNAPTD*_ij_* threshold, the main conclusions of this study remained consistent within the threshold range of 0.05–0.15: cardiomyocyte clusters exhibited local synchronized subgroup structures, and neighboring clusters tended to show higher synchrony. Therefore, we considered PeriodAwareNAPTD*_ij_* ≤ 0.10 to be an operational threshold with a clear time-scale interpretation and robust results for identifying highly synchronized cardiomyocyte cluster pairs.

### 3.5. Extension to Three-Dimensional Engineered Cardiac Tissues

The experimental validation in this study was primarily based on two-dimensional bright-field microscopy videos. Therefore, the current framework is mainly applicable to cardiomyocyte cluster segmentation, beating signal extraction, and synchrony analysis under two-dimensional culture conditions. Nevertheless, the core concept of this framework, including microscopy image or video input, target region segmentation, dynamic signal extraction within regions of interest, time-series beating analysis, and construction of cardiomyocyte cluster synchrony networks, has the potential to be extended to three-dimensional engineered cardiac tissue models. For three-dimensional culture systems that more closely resemble native cardiac tissue, the input data would no longer be two-dimensional microscopy videos, but rather 3D/4D volumetric time-series data acquired via z-stack, multi-view, or other three-dimensional microscopy modalities. Therefore, future applications would require further extension of the current two-dimensional segmentation network to volumetric segmentation models, such as 3D U-Net [30], voxel-based segmentation networks, or 2.5D/4D spatiotemporal models that integrate slice-level features with temporal information. Meanwhile, the ROI used in two-dimensional analysis would also need to be extended to a three-dimensional volume of interest (VOI), combined with voxel-level distance transformation and three-dimensional watershed methods, to enable segmentation and tracking of three-dimensional cardiomyocyte clusters or tissue structures.

In addition, in three-dimensional cardiac tissues, beating behavior is no longer manifested only as grayscale or morphological changes within a two-dimensional plane, but also involves multiple mechanical behaviors. Therefore, if this framework is applied to three-dimensional scaffolds or engineered cardiac tissue constructs, future studies may need to integrate motion field reconstruction with electrophysiological signal monitoring to more accurately capture electromechanical activity [31]. For three-dimensional engineered cardiac tissues, this framework may be used to assess the spatial organization of cardiomyocytes within scaffold materials, the beating process, and the formation of local synchrony networks. For polymer microcarrier-assisted cardiomyocyte transplantation, the framework may further be used to analyze whether stable synchronous beating relationships are established between transplanted cardiomyocyte clusters and host myocardium, thereby providing a potential tool for non-invasive and quantitative assessment of graft-host electromechanical coupling.

## 4. Conclusions

This study proposes and validates a label-free intelligent framework for analyzing cardiomyocyte cluster beating videos. The framework targets two key challenges: accurate segmentation for dense clusters and quantitative characterization for their collective beating performance and synchrony. We built an in-house cardiomyocyte dataset and developed CardioSegNet, a multi-task deep learning model that integrates attention mechanisms with three prediction heads (semantic, contour, and distance transform), followed by a watershed-based post-processing strategy. This design improved segmentation accuracy, robustness, and boundary alignment. We then applied a reference-frame pixel-difference algorithm to video streams to extract time-series beating signals for each cluster. Based on these signals, we performed multi-parametric functional characterization and synchrony analysis across multiple cluster instances. Notably, the PeriodAwareNAPTD*_ij_* metric employed in this study enables systematic evaluation of coordination patterns and spatial heterogeneity in the cluster network. The results show that cluster beating is not globally uniform, but organized into distinct subgroups. Inter-cluster synchrony showed a weak association with spatial distance, with nearby clusters tending to be more synchronized, suggesting a stratified population structure and spatial heterogeneity.

The computational framework developed in this study addresses a key limitation of earlier methods: the difficulty of segmenting adherent cell clusters. It reconstructs the macroscopic beating state of cardiomyocytes and enables a realistic assessment of beating behavior at the cluster level. This makes it useful for large-scale cardiovascular drug screening, in vitro disease modeling, and early cardiotoxicity assessment. Several directions for future improvement remain. First, the current image-processing pipeline was designed primarily for two-dimensional microscopy videos; therefore, its validation remains focused on cardiomyocyte cluster analysis under two-dimensional culture conditions. Future work could further extend this framework to three-dimensional or four-dimensional imaging data for three-dimensional cardiomyocyte cluster segmentation, tissue deformation tracking, and the analysis of complex cardiac organoids. Second, cardiomyocyte cultures often contain adhesion with mononuclear cells and fibroblasts, which can interfere with segmentation and identification. Future models should aim to distinguish and segment these three cell types more accurately for higher-precision cardiomyocyte segmentation. Third, beating intensity in this study is described using normalized differences, so the result is a relative measure. Future work could integrate cell mechanics and map the normalized signal to directly interpretable mechanical parameters such as contractile force or displacement, turning relative comparison into a quantitative description with absolute biophysical meaning.

## Figures and Tables

**Table 1:** Quantitative comparison results of the three segmentation methods on the test set.

Model	Dice ⬆ ^1^	HD95 (µm) ⬇ ^1^
CardioSegNet	0.8868 ± 0.0617	93.02
U-Net	0.8752 ± 0.0651	99.16
DeepLabV3+	0.8585 ± 0.0704	113.15
Cellpose-SAM (zero-shot)	0.8026 ± 0.1139	135.82

^1^ ⬆ indicates higher is better; ⬇ indicates lower is better.

**Table 2:** Definitions of single-cell beating signal parameters.

Parameter	Symbol	Definition
Amplitude	*A_i_*	Signal difference between the main peak and the preceding trough.
Beating period	*T_i_*	Time interval between two adjacent main peaks.
Beating rate	BPM	Number of beats per minute, calculated as BPM = 60/*T_i_*.
Rise time	*T*_r,*i*_	Time difference from the preceding trough to the main peak.
Decay time	*T*_d,*i*_	Time difference from the main peak to the following trough.
Half-amplitude width	TIBD_50,*i*_	Duration of the waveform at 50% of the beat amplitude.
Beat rate irregularity	BRI	Coefficient of variation in beating periods.

**Table 3:** Beating parameters of individual cells in Figure 5a (see Videos S3 and S4 in Supplementary Material for original video).

Parameter	Sample 1	Sample 2	Sample 3
Beating period (s)	1.118571	1.069091	1.066818
Beating rate (bpm)	53.6398	56.1224	56.2420
BRI	0.275082	0.210216	0.180801

## Data Availability

The data presented in this study are available on request from the corresponding author due to privacy.

## Supplementary Materials

The following supporting information can be downloaded at: https://www.mdpi.com/article/10.3390/bioengineering13070742/s1, Figure S1: Examples of beating curve pairs for highly synchronized cell clusters where PeriodAwareNAPTD*_ij_* ≤ 0.10 can be determined; Figure S2: Cardiomyocyte Cluster Functional Characterization and Monitoring Platform; Figure S3: Reconstructed beating curves of multiple cell clusters in Video S3; Figure S4: Relationship between PeriodAwareNAPTDij and peak-sequence Pearson correlation; Figure S5: Relationship curves between the physical distance and the synchrony metric PeriodAwareNAPTD*_ij_* for cell clusters in Videos S1, S7, S8, S13, S15 and S16; Figure S6: Synchrony subnetwork graphs of four instances: Video S1, Video S4, Video S7 and Video S8; Table S1: Summary of correlations between PeriodAwareNAPTD*_ij_* and peak-sequence Pearson correlation across Videos S1–S16; Table S2: Summary of Pearson and Spearman correlation analyses between PeriodAwareNAPTD*_ij_* and inter-cluster spatial distance across Videos S1–S16; Table S3: Sensitivity analysis of highly synchronized cluster-pair proportions under different PeriodAwareNAPTD*_ij_* thresholds across Videos S1–S16; Table S4. Sensitivity analysis of identified subgroup structures under different PeriodAwareNAPTD*_ij_* thresholds across Videos S1–S16; Table S5: Mean spatial distances of synchronous and asynchronous cardiomyocyte cluster pairs under different PeriodAwareNAPTD*_ij_* thresholds; Videos S1–S16: Representative beating recordings of 16 cardiomyocyte clusters.

## Abbreviations

The following abbreviations are used in this manuscript:

ROI	Region of Interest
BRI	Beat rate irregularity
