# Supplementary material for: A Multi-Task Deep Learning Framework for Characterizing Beating Behavior and Synchrony in Cardiomyocyte Clusters
Source: Bioengineering (Basel). 2026 Jun 25;13(7):742. doi: 10.3390/bioengineering13070742 (PMC13405318; doi:10.3390/bioengineering13070742)
Supplement: Supplementary file 1 [file bioengineering-13-00742-s001.zip › bioengineering-4341418-supplementary.pdf]

Supplementary Materials

A Multi-Task Deep Learning Framework for Characterizing Beating Behavior and Synchrony in Cardiomyocyte Clusters

Tianxin Wang[1], Xinjie Liu[2], Fangshuo Zhang[1], Qianwen Guo[3], Xiaoyu Li[4], Yuanyuan Sun[3,*] and Jingjing Xu[1,*]

1 School of Integrated Circuits, Shandong University, Jinan 250100, China

2 School of Software, Shandong University, Jinan 250100, China

3 State Key Laboratory for Innovation and Transformation of Luobing Theory; Key Laboratory of Cardiovascular Remodeling and Function Research of MOE, NHC, CAMS and Shandong Province; Department of Cardiology, Qilu Hospital of Shandong University, Jinan, China, 250012

4 School of Clinical Medical Sciences, Clinical medicine, Cheeloo College of Medicine, Shandong University, Jinan, China, 250012

* Co-corresponding authors: xujj@sdu.edu.cn, yuanyuansun@sdu.edu.cn

Contents

Figure S1. Examples of beating curve pairs for highly synchronized cell clusters where PeriodAwareNAPTD$_{ij}$ ≤ 0.10 can be determined. 3

Figure S2. Cardiomyocyte Cluster Functional Characterization and Monitoring Platform. 4

Figure S3. Reconstructed beating curves of multiple cell clusters in Video S3. 6

Figure S4. Relationship between PeriodAwareNAPTDij and peak-sequence Pearson correlation 8

Figure S5. Relationship curves between the physical distance and the synchrony metric PeriodAwareNAPTD$_{ij}$ for cell clusters in Videos S1,S7,S8,S13,S15,S16. 9

Figure S6. Synchrony subnetwork graphs of four instances: Video S1, Video S4, Video S7, and Video S8. 10

Table S1. Summary of correlations between PeriodAwareNAPTD$_{ij}$ and peak-sequence Pearson correlation across Videos S1-S16. 12

Table S2. Summary of Pearson and Spearman correlation analyses between PeriodAwareNAPTD$_{ij}$ and inter-cluster spatial distance across Videos S1-S16. 13

Table S3. Sensitivity analysis of highly synchronized cluster-pair proportions under different PeriodAwareNAPTD$_{ij}$ thresholds across Videos S1-S16. 14

Table S4. Sensitivity analysis of identified subgroup structures under different PeriodAwareNAPTD$_{ij}$ thresholds across Videos S1-S16. 15

Table S5. Mean spatial distances of synchronous and asynchronous cardiomyocyte cluster pairs under different PeriodAwareNAPTD$_{ij}$ thresholds 17

Videos S1–S16. Representative beating recordings of 16 cardiomyocyte clusters. 18

Figure S1

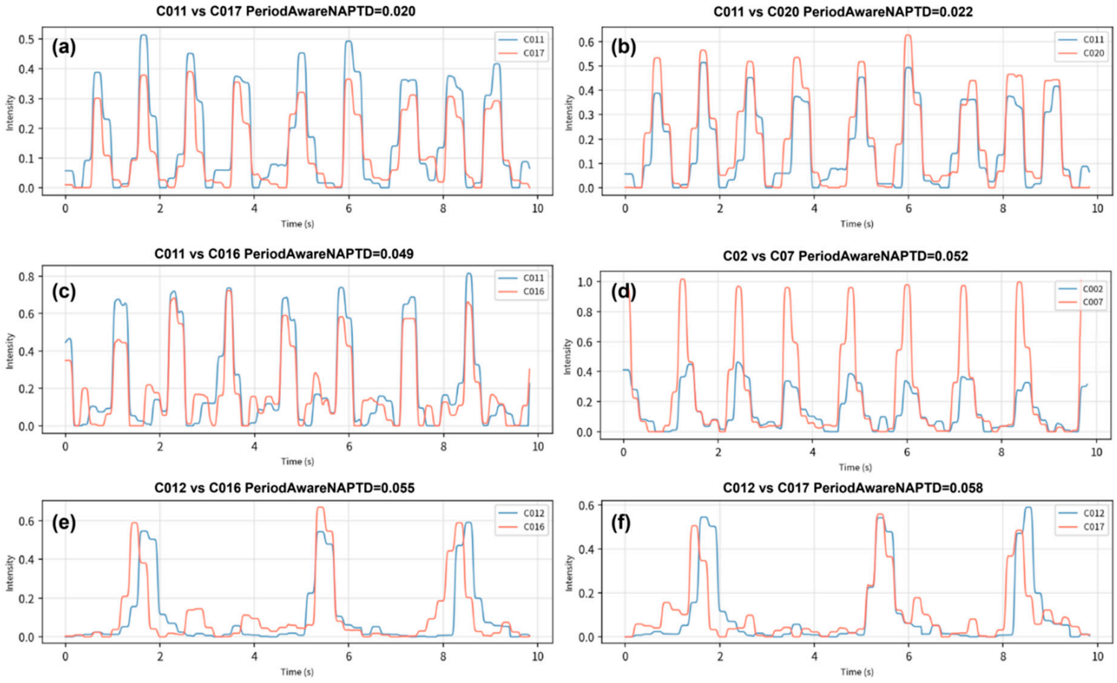

Figure S1. Examples of beating curve pairs for highly synchronized cell clusters where $\text{PeriodAwareNAPTD}_{ij} \leqslant 0.10$ can be determined. (a), (b), (c), (d), (e), (f) are arranged in ascending order of $\text{PeriodAwareNAPTD}_{ij}$ values. It can be observed that the smaller the $\text{PeriodAwareNAPTD}_{ij}$ value of a cell cluster pair, the higher the degree of overlap between their corresponding beating curves and the better the synchrony; conversely, the larger the $\text{PeriodAwareNAPTD}_{ij}$ value, the lower the degree of overlap and the worse the synchrony.

Figure S2

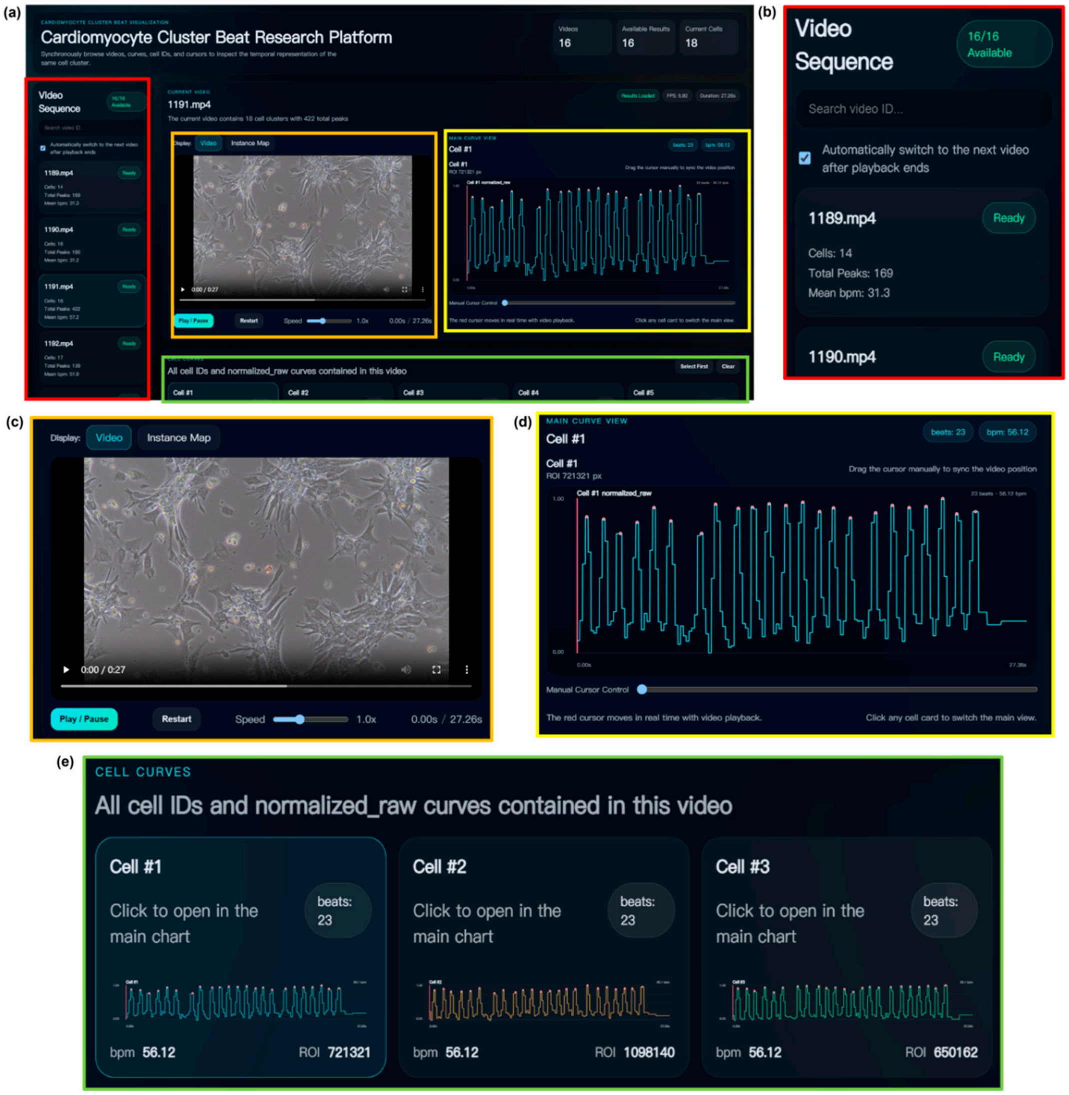

Figure S2. Cardiomyocyte Cluster Functional Characterization and Monitoring Platform. **(a)** Overall platform interface, supporting users to upload their own recorded videos of cardiomyocyte cluster beating for analysis; **(b)** Video selection list; **(c)** Video playback window, where users can choose between the video view or the segmentation mask view. After selecting a cell cluster curve to observe, the video will outline the corresponding cell cluster and display a marker at the frame timing of the beating peak for user observation; **(d)** Output window for the beating

curve of the selected cell cluster, with a cursor that supports manual sliding to any frame time; **(e)** All detected cell clusters under this video and their corresponding output beating curves, automatically listing the number of detected beating peaks and the BPM (beats per minute) of the cell cluster.

Figure S3

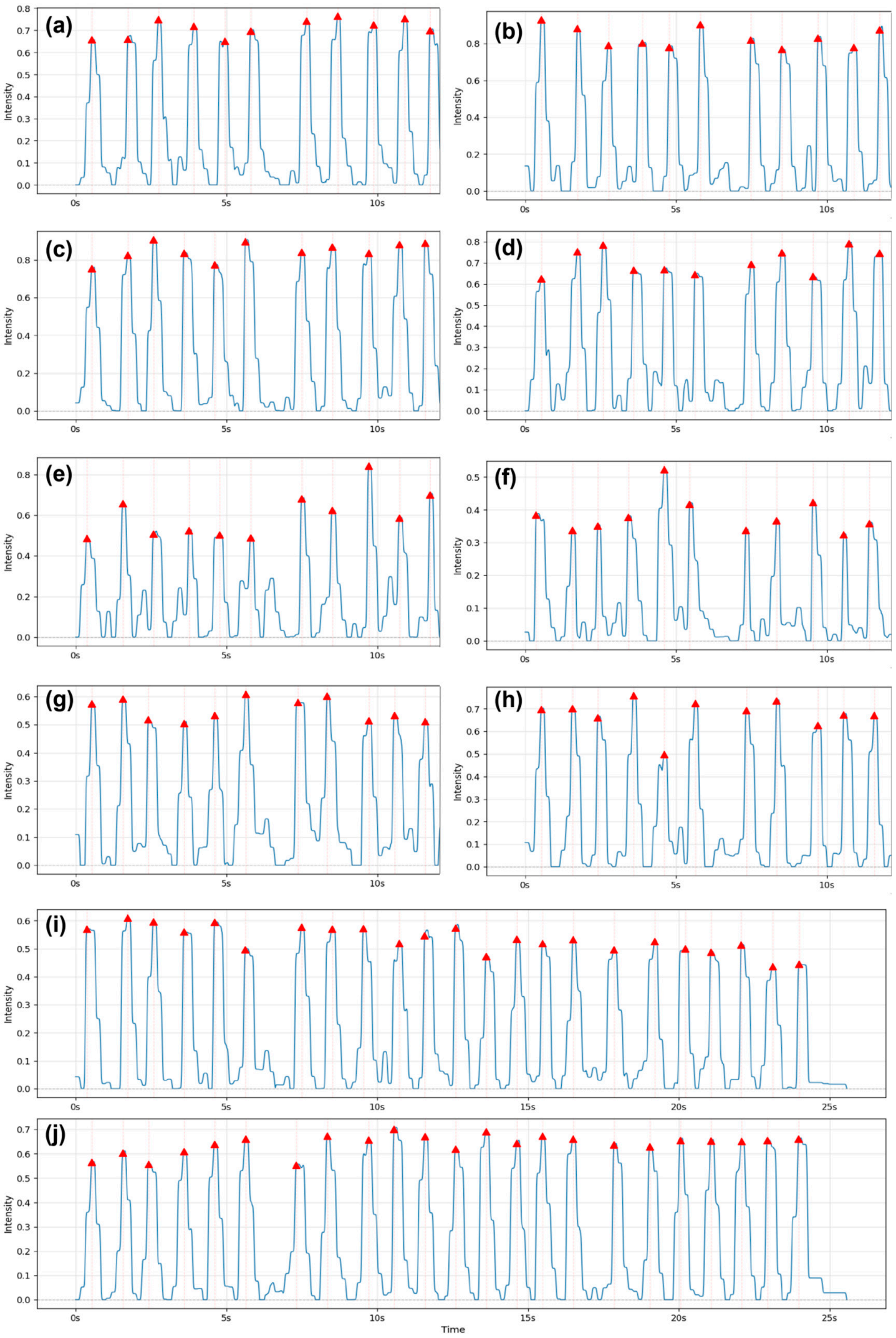

Figure S3. Reconstructed beating curves of multiple cell clusters in Video S3. **(a)**

Sample 1; **(b)** Sample 2; **(c)** Sample 3; **(d)** Sample 4; **(e)** Sample 5; **(f)** Sample 6; **(g)** Sample 7; **(h)** Sample 8; **(i)** Sample 9; **(j)** Sample 10.

Figure S4

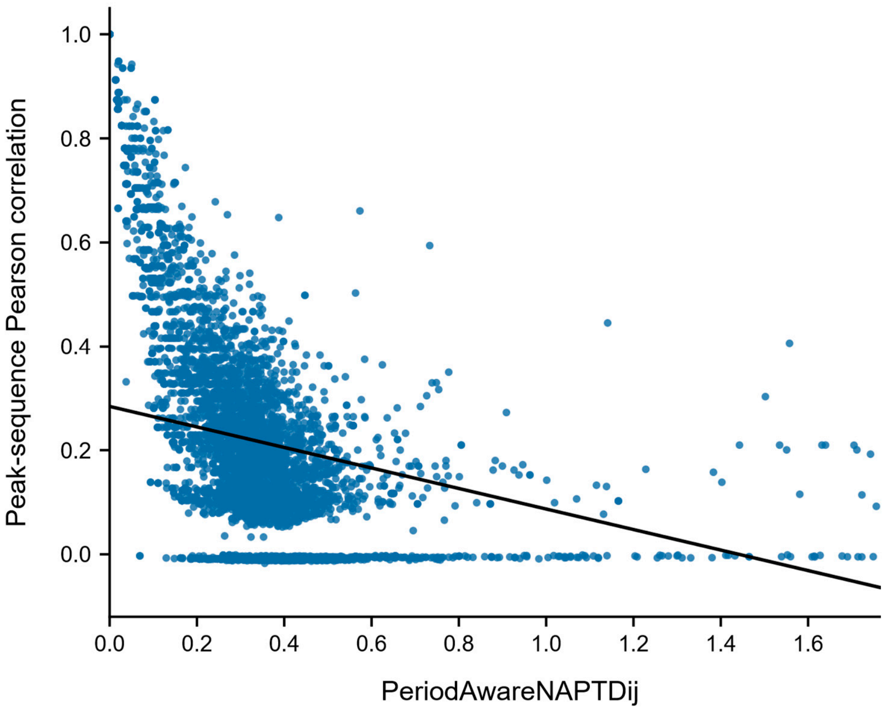

Figure S4. Relationship between PeriodAwareNAPTDij and peak-sequence Pearson correlation

Figure S5

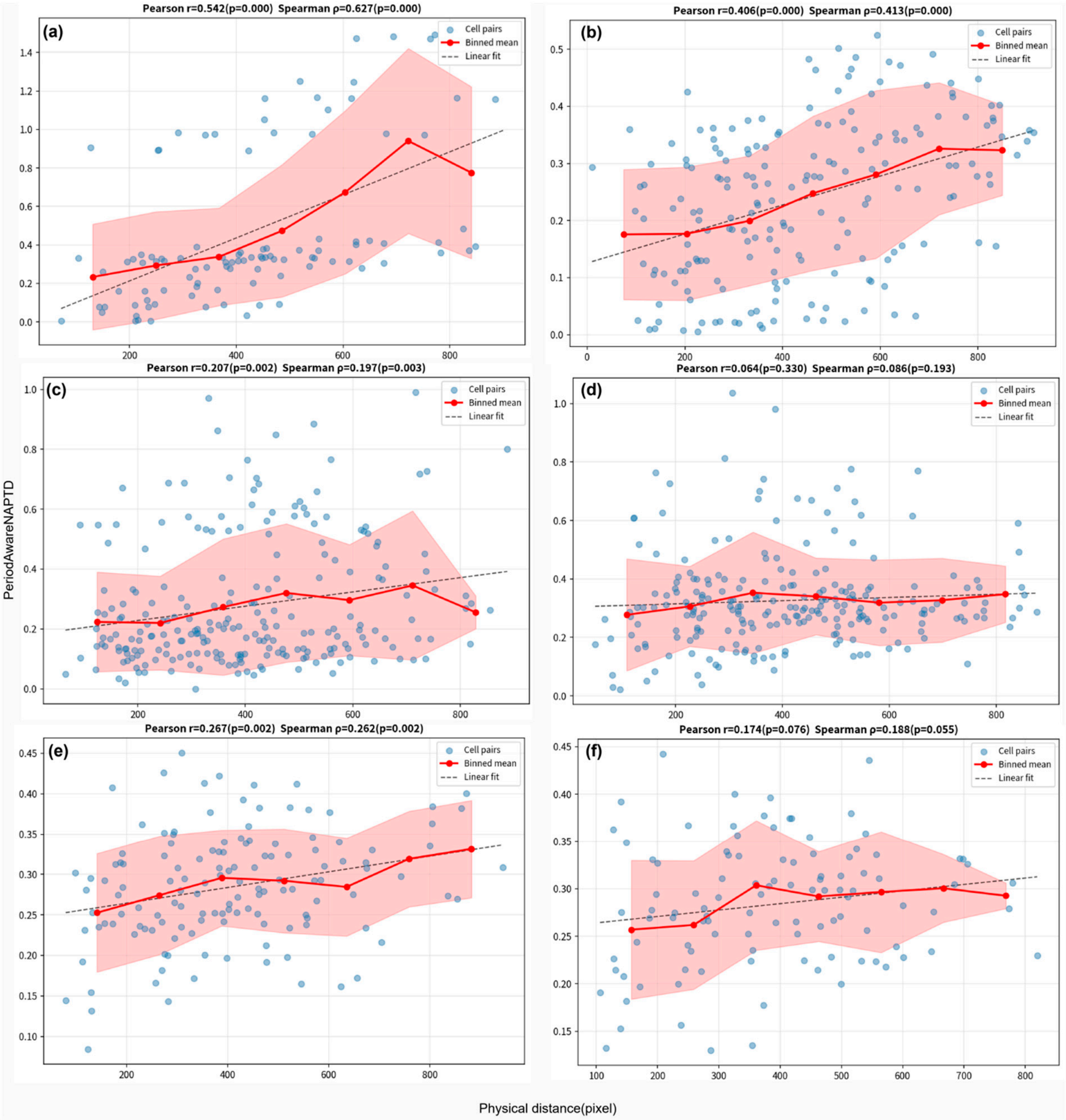

Figure S5. Relationship curves between the physical distance and the synchrony metric $\mathrm{PeriodAwareNAPTD}_{ij}$ for cell clusters in Videos S1,S7,S8,S13,S15,S16. **(a)** Curve corresponding to the instance of Video S1; **(b)** Curve corresponding to the instance of Video S7; **(c)** Curve corresponding to the instance of Video S8; **(d)** Curve corresponding to the instance of Video S13; **(e)** Curve corresponding to the instance of Video S15; **(f)** Curve corresponding to the instance of Video S16.

Figure S6

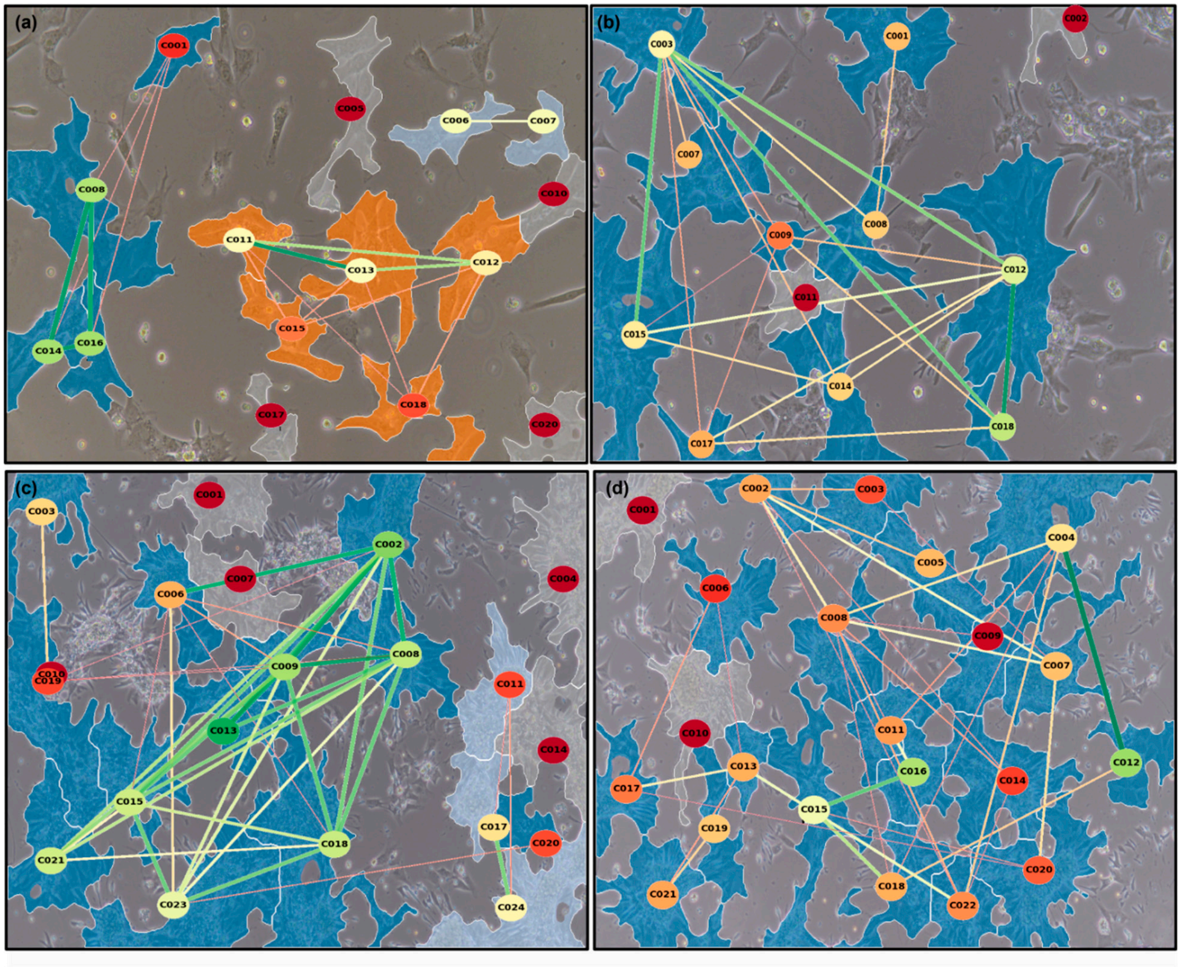

Figure S6. Synchrony subnetwork graphs of four instances: Video S1, Video S4, Video S7, and Video S8. **(a)** Synchrony subnetwork graph of Video S1; **(b)** Synchrony subnetwork graph of Video S4; **(c)** Synchrony subnetwork graph of Video S7; **(d)** Synchrony subnetwork graph of Video S8. Cell clusters of the same color (excluding gray) form a subnetwork. Gray indicates cells that are detected to have beating behavior but have a PeriodAwareNAPTD$_{ij}$ > 0.10 with all other cells in the instance, meaning they do not exhibit synchronous beating with any other cell. The color of the line between two cell clusters represents the degree of synchrony between them. The color

of a cell cluster node represents its BRI parameter value, which indicates the regularity of the rhythm.

Table S1. Summary of correlations between PeriodAwareNAPTD$_{ij}$ and peak-sequence Pearson correlation across Videos S1-S16.

Video	Pearson Correlation (r)[1]	p value for Pearson correlation	Spearman's rank correlation (ρ)[2]	p value for Spearman correlation
S1	-0.5989	0.0001	-0.5990	0.0001
S2	-0.3453	0.0006	-0.3822	0.0002
S3	-0.8962	0.0001	-0.9200	0.0001
S4	-0.8247	0.0001	-0.8168	0.0001
S5	-0.5745	0.0001	-0.5938	0.0001
S6	-0.8251	0.0001	-0.7554	0.0001
S7	-0.7694	0.0001	-0.7131	0.0001
S8	-0.6107	0.0001	-0.5759	0.0001
S9	-0.7695	0.0001	-0.7093	0.0001
S10	-0.6225	0.0001	-0.5630	0.0001
S11	-0.6256	0.0001	-0.5815	0.0001
S12	-0.4576	0.0001	-0.5185	0.0001
S13	-0.7608	0.0001	-0.6045	0.0001
S14	-0.3881	0.0001	-0.4422	0.0001
S15	-0.3123	0.0001	-0.5868	0.0001
S16	-0.6907	0.0001	-0.5875	0.0001

[1] $\bar{r} \pm \mathrm{std} = -0.6295 \pm 0.1791$

[2] $\bar{\rho} \pm \mathrm{std} = -0.6218 \pm 0.1347$

Table S2. Summary of Pearson and Spearman correlation analyses between PeriodAwareNAPTD$_{ij}$ and inter-cluster spatial distance across Videos S1-S16.

Video	Pearson Correlation (r)[1]	p value for Pearson correlation	Spearman's rank correlation (ρ)[2]	p value for Spearman correlation
S1	0.542	0.0001	0.627	0.0001
S2	0.036	0.4348	0.333	0.2745
S3	0.131	0.0041	0.202	0.0086
S4	-0.08	0.6347	-0.106	0.6717
S5	0.032	0.0749	0.132	0.6327
S6	0.638	0.0001	0.600	0.0001
S7	0.406	0.0001	0.413	0.0001
S8	0.207	0.0002	0.197	0.0002
S9	0.106	0.0001	0.128	0.0001
S10	0.044	0.4307	0.058	0.0422
S11	0.045	0.0752	0.051	0.1993
S12	0.097	0.0001	0.111	0.0100
S13	0.064	0.0005	0.086	0.0354
S14	0.166	0.0070	0.186	0.3543
S15	0.267	0.0001	0.262	0.0001
S16	0.174	0.0002	0.188	0.0006

[1] $\bar{r} \pm \text{std} = 0.180 \pm 0.196$

[2] $\bar{\rho} \pm \text{std} = 0.217 \pm 0.195$

Table S3. Sensitivity analysis of highly synchronized cluster-pair proportions under different PeriodAwareNAPTD$_{ij}$ thresholds across Videos S1-S16.

Video	0.05	0.1	0.15
S1	0.087912	0.164835	0.186813
S2	0.008333	0.008333	0.025
S3	0.24183	0.405229	0.601307
S4	0.161765	0.316176	0.610294
S5	0.012987	0.038961	0.073593
S6	0.082011	0.179894	0.328042
S7	0.055385	0.147692	0.224615
S8	0.059289	0.12253	0.264822
S9	0.017204	0.070968	0.144086
S10	0.002151	0.017204	0.027957
S11	0	0.003565	0.016043
S12	0.002016	0.004032	0.010081
S13	0.01087	0.061594	0.148551
S14	0.002646	0.013228	0.02381
S15	0	0.004926	0.017241
S16	0.003333	0.026667	0.06

Table S4. Sensitivity analysis of identified subgroup structures under different PeriodAwareNAPTD$_{ij}$ thresholds across Videos S1-S16.

Video	0.05	0.1	0.15
S1	{C001,C004,C006, C014,C022}; {C002,C003,C011}	{C001,C004,C006,C007,C014, C022}; {C002,C003,C011}	{C001,C004,C006,C007,C014, C022}; {C002,C003,C011}
S2	{C001,C002}	{C001,C002}	{C001,C002}; {C004,C005}; {C013,C017}
S3	{ C06, C012, C013, C015, C016, C017}; {C003, C004};	{ C06, C010, C012, C013, C014, C015, C016, C017}; {C003, C004, C011}; {C001, C002, C005}	{C003, C004, C011 C06, C010, C012, C013, C014, C015, C016, C017}; {C001, C002, C005}
S4	{C001,C002,C004, C005,C006,C007, C009,C010,C017, C019}	{C001,C002,C003,C004,C005, C006,C007,C009,C010,C012, C017,C018,C019}	{C001,C002,C003,C004,C005, C006,C007,C009,C010,C011, C012,C013,C017,C018,C019}
S5	{C003,C018,C022}	{C003,C018,C019,C022,C024, C032}; {C002,C005}; {C009,C015}	{C003,C018,C019,C022,C024, C032}; {C004,C009,C015,C030}; {C002,C005,C035}
S6	{C001,C004,C008, C009,C012,C015, C017,C018,C020, C023}; {C005,C006,C007, C010,C019,C021, C029}	{C001,C004,C005,C006,C007, C008,C009,C010,C011,C012,C014,C015,C017,C018,C019,C020,C021,C023,C025,C029}	{C001,C003,C004,C005,C006, C007,C008,C009,C010,C011, C012,C014,C015,C016,C017, C018,C019,C020,C021,C023, C025,C027,C028,C029}
S7	{C001,C003,C012, C014,C022}; {C010,C013,C017, C021,C024}; {C006,C011,C016}	{C001,C003,C006,C008,C011, C012,C014,C016,C018,C020,C022,C027,C030}; {C010,C013,C017,C021,C024}	{C001,C002,C003,C006,C008, C009,C011,C012,C014,C016,C018,C020,C022,C027,C030}; {C010,C013,C017,C021,C024}
S8	{C001,C006,C009, C010,C020,C024, C026}; {C004,C005,C022}; {C013,C017}; {C015,C016}	{C001,C006,C009,C010,C018, C020,C024,C026,C028}; {C004,C005,C012,C022}; {C008,C015,C016}; {C013,C017}	{C001,C004,C005,C006,C007, C009,C010,C012,C018,C020, C022,C024,C026,C028}; {C008,C014,C015,C016}; {C013,C017,C019}

S9	{C012,C014,C016, C017}; {C007,C021}; {C009,C029}	{C002,C006,C011,C012,C013, C014,C016,C017,C023,C024, C033}; {C007,C021,C030}; {C009,C029}; {C025,C027}	{C002,C006,C011,C012,C013, C014,C016,C017,C023,C024, C025,C026,C027,C032,C033}; {C007,C021,C030}; {C001,C035}; {C003,C031}; {C009,C029}
S10	{C009,C023}	{C006,C009,C023,C028}; {C002,C007,C013}; {C001,C005}	{C002,C007,C011,C013,C030; {C006,C009,C016,C023,C028) {C001,C005}; {C020,C026}
S11	None[1]	{C002,C009,C010}	{C002,C008,C009,C010,C017, C033}; {C026,C028,C031}; {C021,C035}
S12	{C002,C007}	{C002,C007}; {C022,C036}	{C005,C022,C032,C036}; {C002,C007}; {C004,C029}
S13	{C002,C017,C030}	{C002,C006,C008,C009,C013, C015,C017,C023,C025,C030}	{C002,C006,C008,C009,C011, C013,C014,C015,C017,C023, C025,C030}; {C010,C020,C027}
S14	{C005,C010}	{C004,C005,C010}; {C008,C027,C033}; {C007,C017}	{C004,C005,C010}; {C008,C027,C033}; {C003,C024}; {C007,C017}; {C011,C014}
S15	None[1]	{C008,C025}; {C014,C020}	{C009,C014,C017,C020}; {C006,C008,C025}; {C007,C023,C030}
S16	{C005,C015}	{C005,C009,C015}; {C001,C012}; {C006,C024}; {C010,C027}; {C011,C029}; {C013,C021}	{C005,C007,C009,C010,C015, C027}; {C006,C008,C023,C024}; {C001,C012}; {C011,C029}; {C013,C021}

[1] None means no synchronous pairs.

Table S5. Mean spatial distances of synchronous and asynchronous cardiomyocyte cluster pairs under different PeriodAwareNAPTD$_{ij}$ thresholds

Video	0.05		0.1		0.15	
	Mean distance of synchronous pairs (px)	Mean distance of asynchronous pairs (px)	Mean distance of synchronous pairs (px)	Mean distance of asynchronous pairs (px)	Mean distance of synchronous pairs (px)	Mean distance of asynchronous pairs (px)
S1	282.82	466.16	287.46	482.13	298.21	484.92
S2	525.67	479.37	525.67	479.37	360.38	482.82
S3	338.29	456.76	350.96	461.68	399.53	465.17
S4	468.80	531.35	467.62	546.02	500.14	554.26
S5	209.12	425.02	198.77	431.28	256.78	435.36
S6	269.31	460.69	297.40	471.34	355.80	577.98
S7	243.24	486.36	315.15	500.23	336.35	512.45
S8	295.51	445.03	313.34	453.32	345.37	468.87
S9	174.76	481.87	236.70	494.91	275.59	509.83
S10	314.33	504.48	248.42	508.55	265.43	510.94
S11	None[1]	464.33	283.13	464.98	213.96	468.42
S12	200.77	465.67	360.36	465.56	438.67	465.40
S13	229.63	506.37	286.23	517.62	383.82	524.22
S14	103.90	481.17	155.39	484.52	178.74	487.52
S15	None[1]	475.67	348.01	476.30	220.99	480.13
S16	188.10	487.98	255.11	493.33	314.20	498.01

[1] None means no synchronous pairs.

Videos S1–S16. Representative beating recordings of 16 cardiomyocyte clusters.

Each video shows the spontaneous contraction of a single cell cluster under standard culture conditions. Video files are named Video_S1.mp4 to Video_S16.mp4 and provided as separate supplementary files.

Supplementary Materials.zip
